# A genome-wide SNP panel for mapping and association studies in the rat

**DOI:** 10.1186/1471-2164-9-95

**Published:** 2008-02-25

**Authors:** Isaäc J Nijman, Sylvia Kuipers, Mark Verheul, Victor Guryev, Edwin Cuppen

**Affiliations:** 1 Hubrecht Institute, Uppsalalaan 8, 3584 CT Utrecht, The Netherlands

## Abstract

**Background:**

The laboratory rat (*Rattus norvegicus*) is an important model for human disease, and is extensively used for studying complex traits for example in the physiological and pharmacological fields. To facilitate genetic studies like QTL mapping, genetic makers that can be easily typed, like SNPs, are essential.

**Results:**

A genome-wide set of 820 SNP assays was designed for the KASPar genotyping platform, which uses a technique based on allele specific oligo extension and energy transfer-based detection. SNPs were chosen to be equally spread along all chromosomes except Y and to be polymorphic between Brown Norway and SS or Wistar rat strains based on data from the rat HapMap EU project. This panel was tested on 38 rats of 34 different strains and 3 wild rats to determine the level of polymorphism and to generate a phylogenetic network to show their genetic relationships. As a proof of principle we used this panel to map an obesity trait in Zucker rats and confirmed significant linkage (LOD 122) to chromosome 5: 119–129 Mb, where the leptin receptor gene (Lepr) is located (chr5: 122 Mb).

**Conclusion:**

We provide a fast and cost-effective platform for genome-wide SNP typing, which can be used for first-pass genetic mapping and association studies in a wide variety of rat strains.

## Background

The laboratory rat has proven to be an important model organism for human disease, physiology, immunology and pharmacology [1,2]. Over the years, selective breeding for disorders has resulted in the establishment of more than 500 inbred lines that allows the study of factors involved in human multi-factorial diseases under controlled experimental conditions [3]. Genetic mapping and identification of components that affect disease-related phenotypes in the rat can be extremely useful to improve insight in the analogous human syndromes [4].

Traditionally, QTL mapping studies in rat (*Rattus norvegicus*) have been limited by the availability of markers and often focused marker development was needed to successfully link genomic regions to a specific trait (for example [5,6]). Genome information has increased dramatically with technological advances [7] and consequently the number of candidate markers has increased significantly. However, due to the phylogenetic relationship of rat inbred strains, large haplotype blocks can be found grouping several SNPs together. The EU framework 6 programme 'STAR, a SNP and haplotype map for the rat', set out to identify these regions [8]. Combined with new genotyping methods, cheap and versatile genome-wide genotyping has come within reach [9]. Here, we describe the design and application of a panel or 820 rat SNPs for the versatile and fast KASPar system (KBiosciences, UK), which uses a competitive allele-specific PCR combined with a FRET quenching reporter oligo. For each SNP, three unlabeled oligos need to be synthesized, but the assay runs as a standard PCR. After amplification using genomic DNA as a template, the fluorophore signals are determined and genotypes are automatically determined. The SNP panel and genotyping information for more than 30 commonly used rat inbred strains reported here forms a versatile basis for the design of first round genetic mapping and association studies in a wide range of rat strains.

## Results and Discussion

### Selection of SNP candidates for assay setup

**Figure 1:**
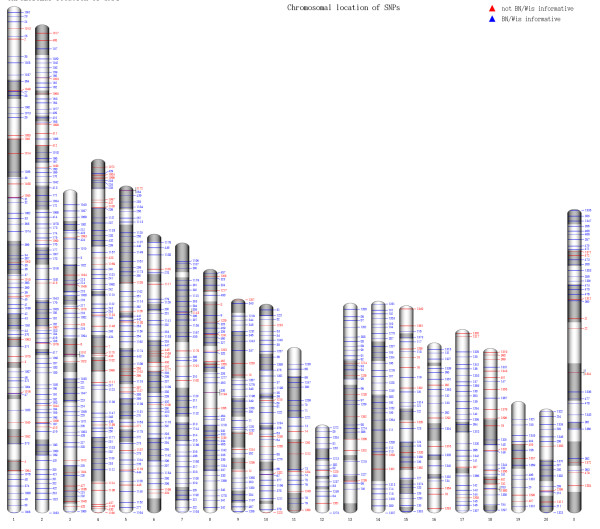
**Chromosomal locations of SNPs on the RSGC3.4 rat genome assembly.** The image was drawn using the idiographica webtool [22]. Giemsa staining is set as chromosomal shading background. Markers indicated in blue were informative between our BN and Wistar animals, and red markers were not, but showed polymorphy in other strains. SNPs in pairwise combinations of strains can be explored at .

Candidate SNPs were selected from various sources (384 and 478 from [8,10] respectively) with a preference for polymorphy between BN and SS or Wistar and an equal physical distribution in the genome. This set was validated in duplo on BN (n = 2), Wistar (n = 2) and BN-Wistar mixed DNA (n = 4) to verify that both alleles could be amplified and heterozygotes are easily discerned from either homozygous genotype. From the originally designed 862 assays, 820 worked reliably (Fig 1) – i.e. showing clear clustering of genotypes- and subsets from these were used for further analyses. The conversion rate of 95% from candidate polymorphism to robust assay is relatively high for a SNP genotyping platform. It should be noted that we did not perform any assay optimization in terms of oligo design or experimental conditions (e.g. numbers of cycles or annealing temperatures) and each assay was run with universal parameters.

### Genotyping

All 820 assays were typed in duplo on a panel of BN (n = 2), Wistar (n = 2), 31 other widely used inbred rat strains that are commercially available, 5 animals from a wild-derived rat strain and 3 wild rats [10]. After removing failed assays, 34.398 duplo genotyping assays remained. From these assays, 49 genotype scores were discrepant between the duplos (0.14%, clustered in 40 SNPs). Another 20 SNPs indicated some heterozygosity while only homozygotes were expected in inbred strains. Additionally, in 1010 cases we could not reliable score one of the duplos (2.9%, clustered in 472 SNPs). In the final dataset, these scores were removed and the final genotype was dictated by the single assay that produced a clear genotype. Overall, 347 SNPs produced perfect duplo genotypes for all the strains without any uncertainty.

**Figure 2:**
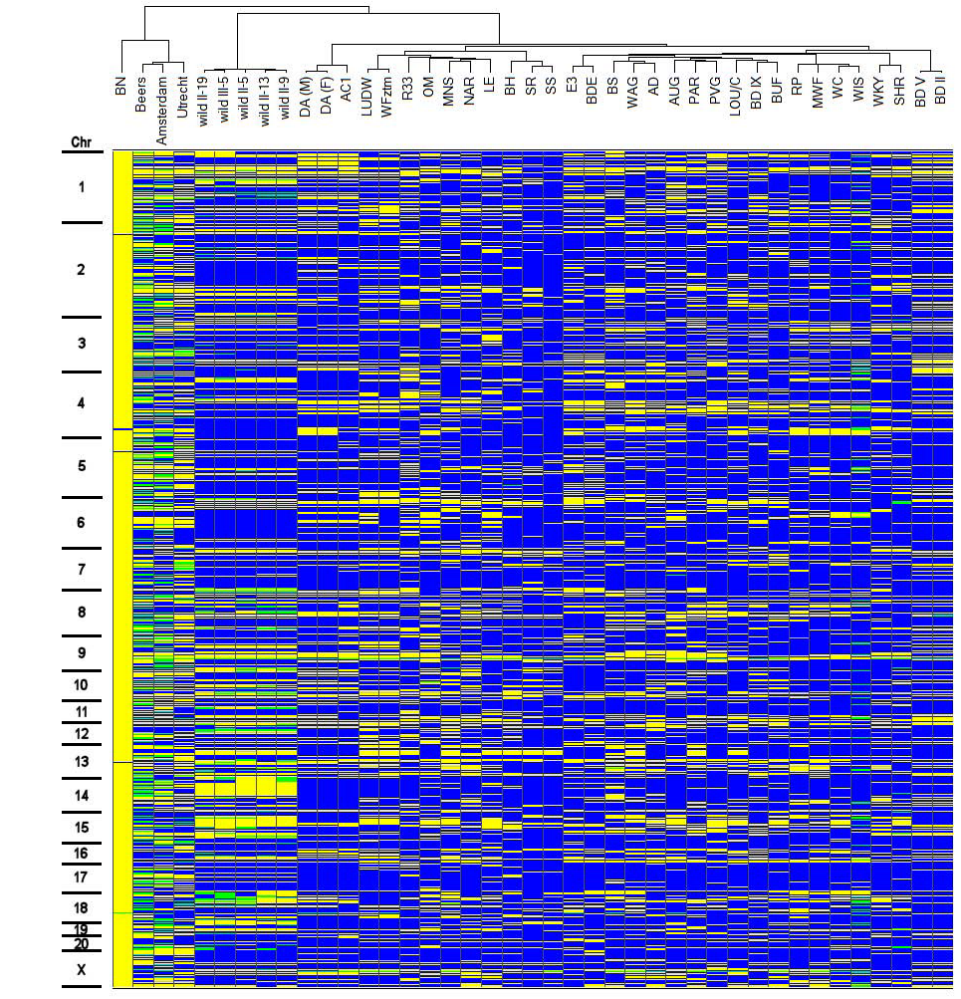
**Heat map of genotyping results of 820 markers in 33 rat strains and 3 wild rats.** Markers are sorted by chromosome and the position of the maker on that chromosome. Yellow indicates BN alleles, blue indicates polymorphic alleles as compared to BN and green positions indicate heterozygous genotypes. A hierarchical cluster tree, based on Wards algorithm is shown on top.

**Figure 3:**
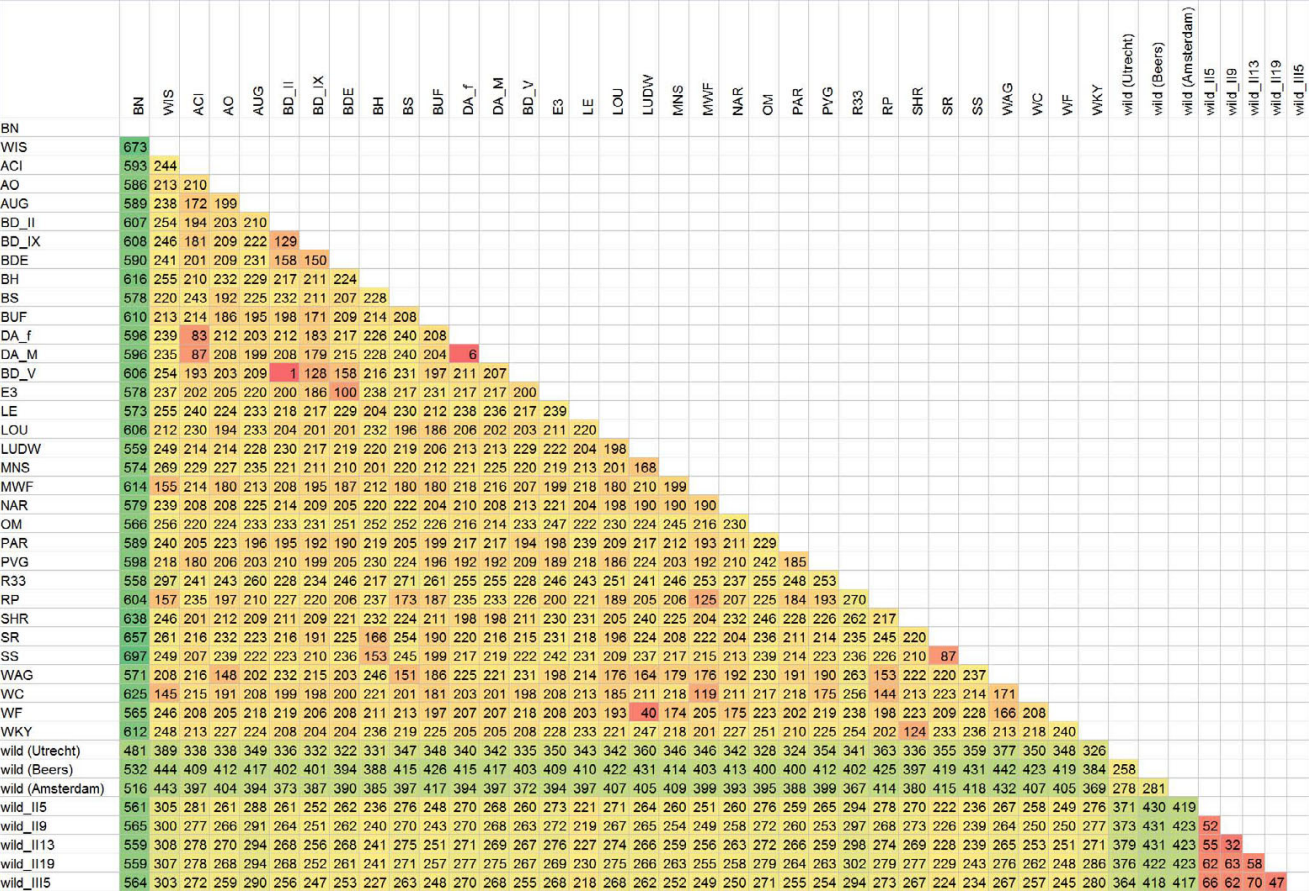
Pairwise comparison of SNP allele differences between strains.

As expected, the genotyping clearly distinguished BN from SS or Wistar (Fig. 2) since SNP design was biased to be polymorphic between these two strains. While the outbred Wistar strain contains much more variable positions, lack of detectable polymorphisms in a few animals can obscure a potential informative marker. Therefore we selected two genome-wide sets of markers. The first panel consists of a set of 632 markers that are polymorphic between BN and the tested Wistar animals. The other markers are not informative in our BN/Wistar crosses, but were chosen to cover big chromosomal gaps where no informative markers were available for BN/Wistar. All the other rat strains show an intermediate pattern with a median number of 231 polymorphic markers for any pairwise strain combination, indicating that this panel is also informative for mapping studies using a wide range of rat strains. Figure 3 shows a pairwise comparison of allele differences between strains. In this case, heterozygote markers are included since they can be informative. In our follow-up study, only the markers with homozygous alleles were used. All genotyping data, including information on the genomic locus and oligonucleotide sequences, can be explored for any arbitrary pairwise strain combination [11].

**Figure 4:**
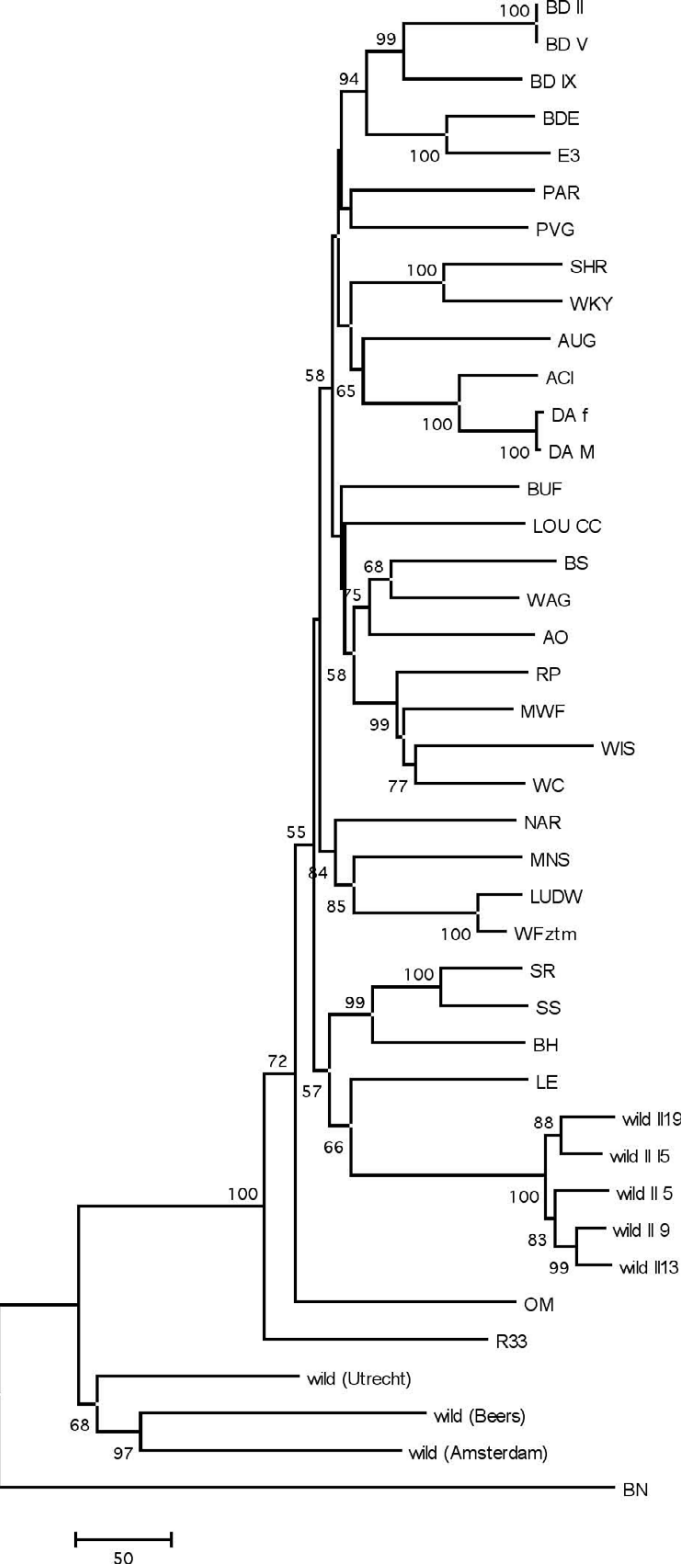
A Neighbor-joining phylogenetic tree of rat strains based on uncorrected p-distances of the genotypes of complete set of 820 markers.

The hierarchical clustering tree (Fig. 4) shows the relationships between the strains and concurs with known genetic background [10] and previous studies [8]. The domesticated wild-derived rats from Groningen (wild II, n = 5) are much more related to each other than the wild rats caught in Utrecht, Amsterdam and Beers, which is most likely due to genetic drift in the colony which is now maintained in the lab for over 30 years.

### Phylogenetic analysis

**Figure 5:**
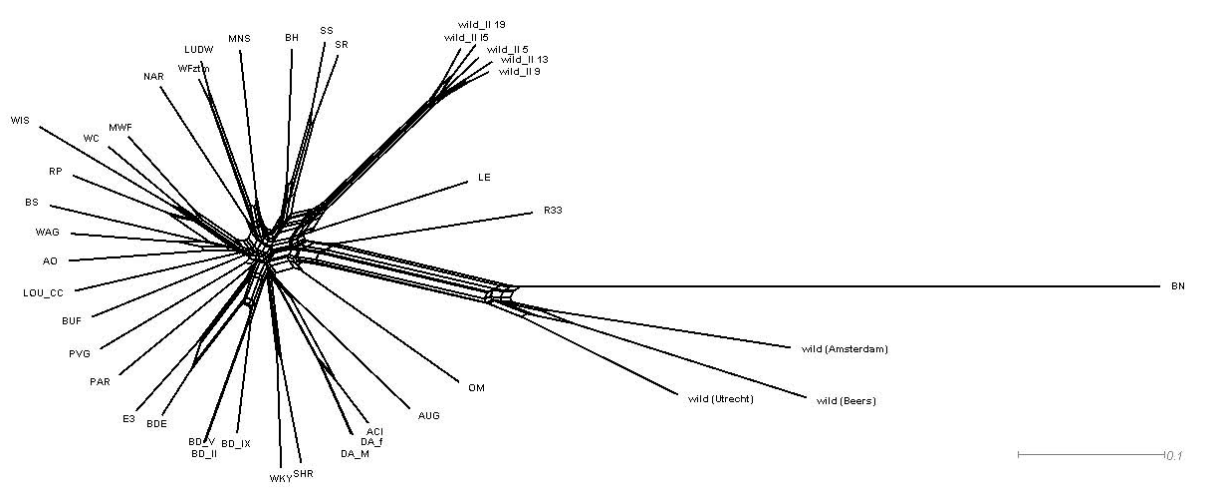
A NeighborNet phylogenetic network of rat strains based on uncorrected p-distances of the genotypes of complete set of 820 markers.

The genotypes of a genome wide SNP panel provide a good data set to infer a phylogenetic structure for this panel of inbred strains. At present, this structure is only available based on a small amount of SNPs [10] or microsatellites [12,13]. The general topology of the hierarchical clustering of Figure 2 is supported by a separate phylogenetic analysis resulting in a Neighbor-Joining tree (Fig. 4). From this tree it is clear that the older nodes are only weakly supported, which is not surprising since a combination of different strains has been used to generate the current inbred strains. Therefore, it is unknown how much each parental strain has contributed to the current strains and a high level of reticulation can be expected. A NeighborNet analysis (Fig. 5) indeed confirms this complex ancestral history and provides a more unbiased view of phylogenetic clusters. From both figures the large branch length towards BN is obvious. This probably reflects the ascertainment bias because SNPs are selected from a comparison with the published genome sequence of the Brown Norway rat [7]. However, similar studies using microsatellite markers, which are less sensitive to this bias, also showed that BN is relatively far diverged from the other rat inbred lines [12,13]. In general, a number of clusters can be distinguished in the unrooted Neighbor-net: 1: SS-like, 2: LUDW-like, 3: Wistar-like, 4: BD-like, 5: WKY/SHR, 6: DA-like and the group containing BN and the wild rats. Most groups are formed by strains that are known to be closely related. For instance in the BD cluster, BDE is an inbred strain crossed from the BD group and E3. Additionally, WKY and SHR are closely related [3] and indeed cluster tightly together. The strains clustering in the Wistar group have known Wistar background [3].

### Mapping of the obese trait in Zucker rats

**Figure 6:**
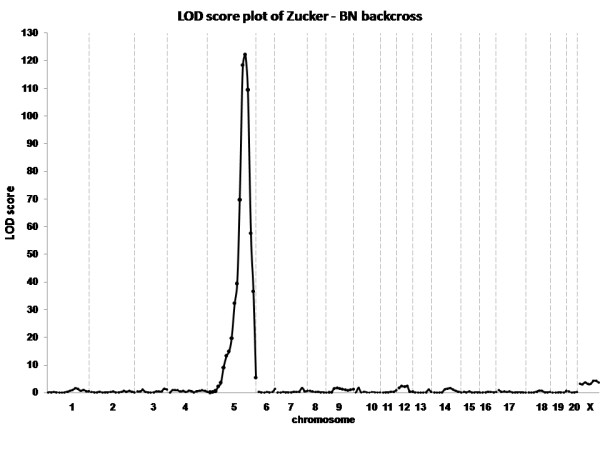
**Linkage analysis graph based on the genotyping results in the Zucker/BN backcross population using 205 informative makers.** A highly significant LOD score for the leptin receptor locus on chromosome 5 is obtained.

As a proof of principle for a mapping study, we used our validated SNP panel in a cross with the Zucker rat strain (originally a cross between Sherman and Merck 13 M strains [14]), a strain which is commonly used as a model in obesity research [15]. For this study, animals from the Zucker strain were outcrossed with Brown Norway and F1 animals were backcrossed with Zucker rats. The resulting BC animals were selected for genotyping based on their obese appearance at 3–4 weeks of age. As our SNP panel was developed to contain polymorphic markers between BN and Wistar or SS (which shares many alleles with Wistar rats [10]), a large number of informative markers was expected. Indeed, 205 out of 547 markers tested were found to be polymorphic and these were genotyped on the 239 animals from the Zucker – BN backcross. After genotyping, the Bn/Wistar mix controls showed a complete heterozygous profile indicating that all alleles were correctly amplified and detected. Genotype calling was successful for 99% of the assays (48,489 out of 48,995). We calculated the LOD scores for the backcross (Fig 6.) and found a single high score locus on chromosome 5 in the region 119–129 Mb. This is in agreement with the fact that the obesity phenotype in Zucker rats is caused by a mutation in the leptin receptor gene (Lepr) on chromosome 5: 122 Mb [16,17]. We show that a panel of 205 markers provides sufficient resolution to map a monogenetic trait to a region of less than 10 Mb. As there are more markers available in the complete panel of 820 validated markers, it is still possible to perform further fine-mapping in regions of interest.

## Conclusion

We established a versatile genome-wide SNP genotyping panel for the rat that is flexible, fast, and relatively cheap (~$0.05 per genotype and only ~$10.00 set up costs per assay). We show that this panel can be used for first round association analysis in genetic linkage and association studies and that it can be applied in a wide range of strain combinations. We show that a known monogenetic trait, like the leptin receptor locus in the Zucker strain, can be easily mapped. Furthermore, in an ongoing ENU screen to generate knockouts in the rat, we mapped a clear phenotype to a region on chromosome 6 with a LOD of 6.5 in a population of 16 affected and 14 unaffected sibs (data not shown). Although this region contains a large number of genes, targeted finemapping with a set of additional Kaspar markers and by sequencing, decreased the size of the linked region and revealed 4 candidate genes. As the rat is increasingly used in genetic studies, tools as described here are expected to further aid in understanding the contribution of genetic variation to phenotypic traits and disease.

## Methods

### Rat samples

We analyzed animals from 34 rat strains, (strain names with RGD ID's between parentheses): ACI/Ztm (10000), AO/OlaHsd (70429), AUG/OlaHsd (67960), BDE/Ztm (67974), BDII/Ztm (67976), BDIX/Ztm (61002), BDV/Ztm (67981), BH/Ztm (67989), BN/Crl (60985), BS/Ztm (68008), BUF/SimRijHsd (60986), DA/Ztm (60997), E3/Ztm (61013), LE/Ztm (60991), LOU/CZtm (68079), LUDW/OlaHsd (68082), MNS/Ztm (60992), MWF/Ztm (68099), NAR/Ztm (737969), OM/Ztm (70452), PAR/Ztm (737940), PVG/OlaHsd (61006), R33/Ztm, RP/AeurRijHsd (68127), SHR (61000), SR/JrHsd (70453), SS/JrHsd (69369), WAG/RijHsd (61008), WC/Ztm, WF/Ztm (61007), WKY/Ztm (61103), outbred Crl:Wistar (10044) and wild/Gro (5 animals wild_11 5–15). Additionally, 3 wild rats caught in Utrecht (wild1/Hubr; 1625284), Amsterdam, and Beers in the Netherlands were analysed.

For the trait mapping analysis we obtained DNA from 239 BC rats which were selected for an obesity phenotype by visual inspection at an age of 3–4 weeks. These animals were derived from a backcross of Zucker/BN F1 heterozygotes with Zucker animals.

### SNP selection and assay design

A Perl script was written to select SNPs from genome and rat HapMap data, evenly spread over the chromosomes and polymorphic between BN and Wistar or SS (Fig 1. [11] and Additional file 1). For a set of 862 SNPs, primers were designed for KASPar genotyping using a tool provided by KBiosciences [18] based on the SNP locus sequence (about 50 nt flanking each side of the SNP are required for the design). The output provides sequence information for two allele-specific oligonucleotides of about 40 nt in length and 1 common oligonucleotide of about 20 nt in length, all of which are standard unmodified and unlabelled oligonucleotides. Detailed information on every marker can be found in Additional file 1 and [11] The three oligonucleotides for each assay were dissolved in 10 mM Tris-HCl (pH 8) to a 100 μM concentration, mixed together as a SNP assay mix (12 μl AS1 + 12 μl AS2 + 30 μl CP + 46 μ; Tris-HCl pH 8) and 2 μl aliquots were distributed into individual wells of 384 well plates by a Tecan Robot (Genesis RSP200 liquid handling workstation including an integrated 96-channel pipetting head TEMO96). Assay plates were frozen at -20°C until use. Each SNP was typed in a total volume of 4 μl in the following reaction mixture: 6 ng DNA, 22 mM MgCl_2_, KTaq, 1 μl 4× reaction mix, 2 μl pre-plated assay mix according to the manufacturer's guidelines (Kbiosciences). Amplification was performed in Applied Biosystems GeneAmp 9700 thermocyclers running the following program: 94°C – 15' then 20 cycles of 94°C-10", 57°C-5" and 72°C-10", followed by 18 cycles of 94°C-10", 57°C-20" and 72°C-40". Fluorescence scanning of the reactions was done in a BMG labtech Pherastar scanner and the results were interpreted by the KlusterCaller 1.1 software (KBiosciences). Per 384 well plate, all SNPs are amplified for a single individual and afterwards all data for each locus is regrouped for all samples by a custom Perl script before interpretation by KlusterCaller.

### Phylogenetic analysis

The phylogenetic analysis of genotypes of the rat strains was performed using the MEGA package [19] and Splitstree [20]. The Neighbour-joining tree and NeighborNet diagram were calculated based on uncorrected p distances. A heat map of SNP data and the hierarchical clustering (Ward's method) tree were drawn by Spotfire Decisionsite 8.2.1 (Tibco, US).

### Mapping the obesity trait

A custom Perl script was written to calculate LOD scores for the obesity trait in the Zucker population. The method used was based on maximum likelihood estimates of the recombination fraction [21]. This script is available upon request.

## Authors' contributions

IN designed the SNP panel, carried out analyses and wrote the manuscript. MV and SK performed Kaspar typings. VG designed part of the panel and assisted in bioinformatic analyses. EC initiated and supervised the project and contributed to the manuscript.

All authors read and approved the final manuscript.

## Supplementary Material

Additional file 1 Supplementary table. All validated KASPar rat SNP markers designed on the BN genome sequence (RGSC 3.4). The map id refers to the graphical representation in Figure 1 (main text). Click here for file
